# Sustainable production of biodiesel from waste cooking oil using magnesium oxide nano catalyst: An optimization study

**DOI:** 10.1038/s41598-024-71930-1

**Published:** 2024-09-24

**Authors:** Sabah Mohamed Farouk, Aghareed M. Tayeb, Randa M. Osman, Shereen M. S. Abdel-Hamid

**Affiliations:** 1 Chemical Engineering Department, Egyptian Academy for Engineering and Advanced Technology (EA&EAT) affiliated to Ministry of Military Production, Km 3 Cairo Belbeis Desert Rd, Cairo, 3066 Egypt; 2 Faculty of Engineering, Minia University, Misr Aswan Agricultural Rd., EL Mahatta, Minya, 2431384 Menia Governorate Egypt; 3 Chemical Engineering and Pilot Plant Department, National Research Centre (NRC), 33 EL Buhouth St, Dokki, 12622 Cairo Governorate Egypt

**Keywords:** Biodiesel, Waste cooking oil, Transesterification, Modelling, MgO nanocatalyst, Environmental sciences, Energy science and technology, Engineering, Nanoscience and technology

## Abstract

Biodiesel is rapidly becoming an efficient substitute for fuel and a potentially significant future renewable energy source. In recent years, used cooking oil has been used as a feedstock for biofuel to reduce production costs. Due to its high catalytic activity, low cost, and eco-friendliness, Nano magnesium oxide (MgO) has attracted attention as a catalyst for biodiesel production. Our work presents the preparation of nanomagnesium oxide (MgO) by the sol–gel method, and its characterization. Optimum conditions and the productive combination of waste cooking oil, methanol, and the synthesized nanocatalyst were predicted using response surface methodology. The optimum conditions were methanol to oil ratio of 7:1, temperature of 50 °C and time of 60 min. The expected values for the yield of biodiesel production responses are quite like the actual values, demonstrating the consistency of the models used for establishing a relationship between the independent process variables and the responses. The predicted model's F-value was 9.09 indicating that the model is significant. The model's pure error had a poor correlation, as the "Lack of Fit F-values” 4.16. The quadratic model fits the data well because the R-squared value for the model equation 92%. The expected values for the yield of biodiesel production responses are quite like the actual values, demonstrating the consistency of the models used for establishing a relationship between the independent process variables and the responses. Biodiesel was characterized using gas chromatography–mass spectrometry and Fourier transform infrared spectroscopy.

## Introduction

Population growth has resulted in a sharp rise in the total number of industries and automobiles, which has led to an ongoing increase in the need for energy. The main drawback of using petroleum-based fuels is the air pollution that petroleum diesel causes^1^. Numerous alternative fuels have been investigated as partial or full replacements for diesel fuel. Given that they are generated in agricultural regions, vegetable oils are suggested as possible diesel substitutes. The oil made from seeds can open options for side work^2^.A fuel made of long-chain fatty acid monoalkyl esters generated from vegetable or animal fats is known as biodiesel^3^ It is relatively simple and has numerous environmental advantages to produce biodiesel from waste vegetable oil. Vegetable oils used for frying produce a lot of waste oil, which could be difficult to get rid of^4^.Their low cost makes them ideal for use in the manufacturing of biodiesel. The optimum starting material for biodiesel production is vegetable oil from plant sources since it has a high conversion rate of pure triglycerides to fatty acid methyl ester and a quick reaction time^5^ Recently, there has been a deep worry about the use of edible vegetable oils and animal fats in the manufacturing of biodiesel since they compete with food ingredients. It is impossible to justify the use of vegetable oils for fuel consumption, such as the creation of biodiesel, given the enormous recent growth in demand for vegetable oils for food. Additionally, the cost of using these oils as fuel may be higher than the price of making biodiesel according to the materials utilised. Waste cooking oil can be considered the least expensive and inexpensive raw material to produce biodiesel among the materials. Chemical or biological catalysts are both used in catalytic transesterification processes. Both homogeneous and heterogeneous catalysts are used in chemical reactions. An acid or alkali catalyst is part of the homogeneous catalyst. The heterogeneous catalyst is made up of solid acid, base, and acid–base bifunctional catalysts as well as biomass waste-based and nanocatalysts^6^ The choice of any catalyst is influenced by the following factors: oil quality, FFA content, operating conditions, necessary catalyst activity, cost, and availability^7^.The focus of the present study is to synthesis MgO nanocatalyst by the precipitation method using MgO nanoparticles and optimise the key transesterification reaction parameters. The catalyst synthesised was used to produce biodiesel from waste cooking oil. The precipitation technique, followed by the calcination method, is used to create highly pure nano-sized magnesium oxide (MgO) catalysts. This nanostructured MgO is characterised by various techniques to explore its physicochemical properties. The efficiency of biodiesel production depends on several factors, including contact time, temperature, and the ratio of methanol to oil. Therefore, the response surface methodology (RSM) model is employed to predict the optimum conditions that influence the production of biodiesel.

## Experimental work

### Materials

Waste cooking oil was initially gathered from sources that were used at home. It was a blend of palm and sunflower oil. Chemicals are utilised without any additional preprocessing. Methanol, magnesium sulphate heptahydrate, and sodium hydroxide were purchased from Alpha Chemika Pharmaceuticals and Chemicals Company, Egypt.

### Methodology

#### Catalyst characterization

##### Morphology by Scanning electron microscope coupled with energy dispersive X-ray scattering scanning

The morphology of the synthesized catalyst was examined by scanning electron microscopy (SEM) with energy dispersive x-ray analysis (EDX) techniques. The scanning electron microscopy (SEM) technique utilizes an electron beam to scan and generate high-resolution electronic images. To enhance comprehension of the elemental composition, the analytical technique of energy-dispersive X-ray spectroscopy (EDX) was employed^8^. SEM and SEM–EDX were recorded on The QUANTA FEG250, located at the National Research Center (NRC) of Egypt.

##### Surface area of catalyst

The surface area of the produced material was calculated using the Brunauer–Emmett–Teller (BET) model. The samples were degassed at 120 °C for the whole night before Using N_2_ adsorption isotherm values. The BET surface area was calculated using QuantachromeNovaWin 3200, USA.

##### Thermo gravimetric analysis

The physico-chemical changes, specifically decomposition or oxidation, were investigated using thermogravimetric analysis (TGA). The thermogravimetric analysis (TGA) was conducted using a TGA Q500 V20.10 instrument. Nitrogen gas was used as the inert medium, flowing at a rate of 50 ml/min. The heating rate was set at 10 °C/min, and the temperature range for the analysis was 25–900 °C at the Science and Technology Centre of Excellence (STCE).

##### X-ray powder diffraction analysis

XRD powder analysis is a widely employed technique in materials science and chemistry for the purpose of determining the chemical composition of a sample by identifying the crystalline phases that are present within it. The crystal-phase structure was analyzed through the utilization of XRD powder technique by employing analytical instruments PAN.t, PERT PRO, and CuKa X-ray radiation ( λ = 1.540A^o^).

#### Analysis of the biodiesel produced

The mass yield was calculated from Eq. (1)^9,10^.

1 $$Mass\; yield\% = \frac{Actual\; Weight\; of\; biodiesel}{Weight\; of\; oil\; used}*100$$

The produced biodiesel's FAME content was measured using (GC). It was applied to evaluate the biodiesel sample's FAME composition. Combinations can be separated via GC analysis depending on their boiling points. The methyl ester retention time values of the isolated fatty acids were calculated using standards of retention times.

Using Eq. (2), we calculated the percentage efficiency with which we converted raw oil into methyl ester.

2 $$Conversion \; efficiency\% \, = \, ester \, content \, \% \, * \, mass \, yield\%$$

This yield was chosen and tested to evaluate the physical and chemical characteristics of biodiesel, including flash point, cloud point, and density according to the American Society for Testing and Materials (ASTM D 6751).

##### Fourier Transform Infrared (FTIR)

Function groups in molecules can be recognized and any interactions-related modifications can be seen with the aid of infrared spectroscopy. Using this method, it is possible to classify molecules based on the infrared wave numbers at which they vibrate. The samples are measured using Class 1 Laser Products (IEC/EN 60,825–1/A2:2001 Avatar Series) in the engineering and advanced technology labs of the Egyptian academy.

##### Gas Chromatography-Mass Spectrometry

An investigation into the biodiesel sample's chemical composition was carried out utilizing a Shimadzu GC 17-A gas chromatograph and a direct capillary column TG WAX-MS with a length of 30 m and a film thickness of 0.25 mm. The temperature in the oven was initially set to 60 ^O^C, where it remained for three minutes before being increased to 300 ^O^C over the course of 24 min.

##### Calorific value

To determine the calorific value of biodiesel, a Parr oxygen bomb calorimeter was utilised. The bomb receives 1 g of material containing extra oxygen at 25 bar^11^. The test is carried out using a Parr 6200 calorimeter at the Science and Technology Centre of Excellence (STCE).

#### Waste cooking oil sample preparation

Waste cooking oil was gathered, filtered to remove food debris, and dried at 110 °C for around 30 min. WCO's density, acid value, and free fatty acid (FFA) value were also assessed.

#### Nano catalyst synthesis

MgO nanoparticles was synthesized by sol–gel technique. Using a magnetic stirrer at 95 degrees Celsius, 45 g of magnesium sulphate heptahydrate was dissolved in 400 ml of distilled water. Drop by drop, 1 M NaOH solution was added to the MgSO_4_.7H_2_O solution for two hours until the pH reached 12.34 (when adding sodium hydroxide solution, white precipitate gradually developed). The metal hydroxide was allowed to sit over night before being dried at 85 °C for a day. After that, the powder was calcined at 650 °C for two hours^12^.

#### Transesterification process

For 10 min a mixture of different methanol to oil ratios (6, 8, and10) and the nano MgO catalyst in accordance with the FFA% in the sample is mixed, At three different temperature settings (30, 50, and 70 °C), 100 ml of used cooking oil was applied^13^ Transesterification progressed as the reaction mixture was continuously stirred for varying lengths of time (60, 90, and 120 min). Atmospheric pressure and a 500-rpm stirring speed were used for all transesterification operations. After the reaction was finished, the mixture was put into a funnel for separation and let to stand for the entire night. Following two cycles for washings in warmed water at 80 ^O^C. then the material was allowed to stand until two layers developed. After the biodiesel was dried, very few suspended solid catalysts were removed by allowing them to settle for two to three days^14^.

### Design of experiments and data processing

Empirical models based on real data are produced using the RSM approach using a combination of statistical and mathematical techniques. For the transesterification reaction in this study, the Box-Behnken method was utilized. To maximize the biodiesel yield, a total of 17 trial runs were carried out. The temperature (between 30 and 70 degrees Celsius), the methanol to oil ratio (between 6 and 10), and the duration (between 60 and 120 min) were the independent process factors that were examined. Evaluating these variables' impact on the biodiesel production process was the aim of this study.

The experimental procedure for the Box-Behnken design is shown in Table 1 along with its levels and variables.

**Table 1:** Experimental variables and levels for Box-Behnken design.

Variables	Symbols	Level		
		– 1	0	+ 1
Time (min)	A	60	90	120
Temperature (^0^C)	B	30	50	70
Methanol to oil (M/O) ratio (mole/mole)	C	6	8	10

## Results and Discussion

### Waste cooking oil characterization

Standard laboratory tests were carried out to determine the chemical and physical characteristics of the waste cooking oil. Table 2 lists the physico-chemical characteristics of the used cooking oil.

**Table 2:** Physico-chemical properties of the used cooking oil.

Physicochemical Properties	Values
Density at 25 °C (g/m^3^)	0.91
Acid value (mg KOH/gm)	1.295
FFA content (Wt % of oil)	0.648

### Catalyst characterization

#### Scanning electron microscope coupled with energy dispersive X-ray scattering Scanning analysis

**Fig. 1:**
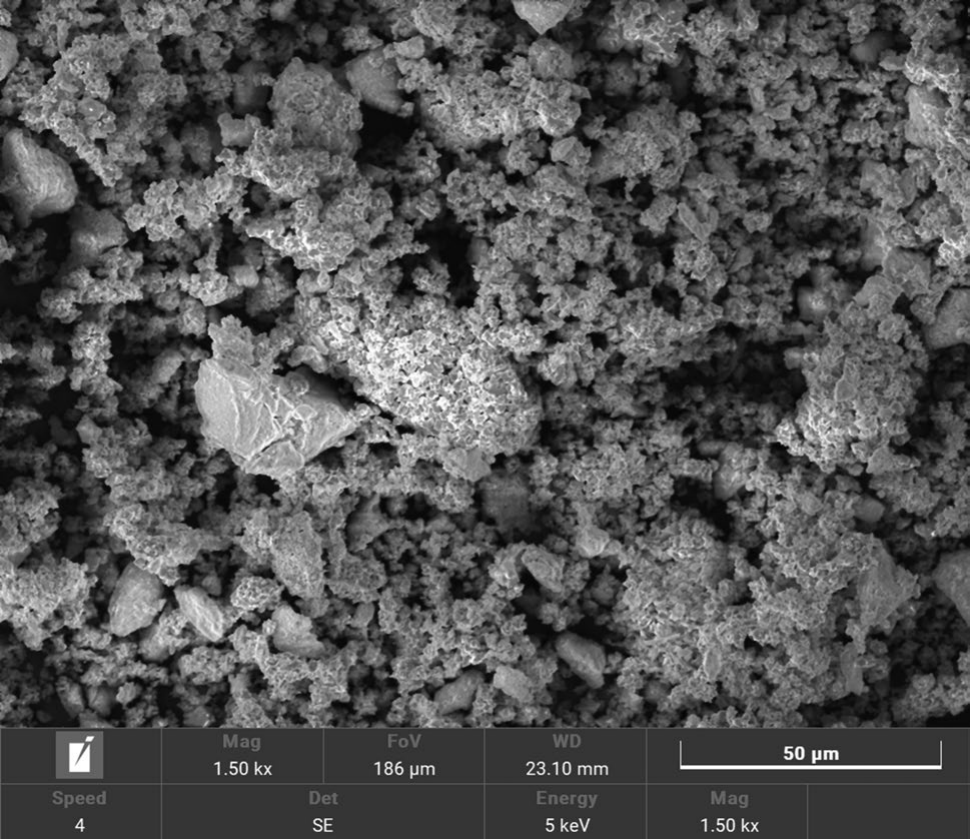
SEM micrographs of MgO nano-catalyst.

**Fig. 2:**
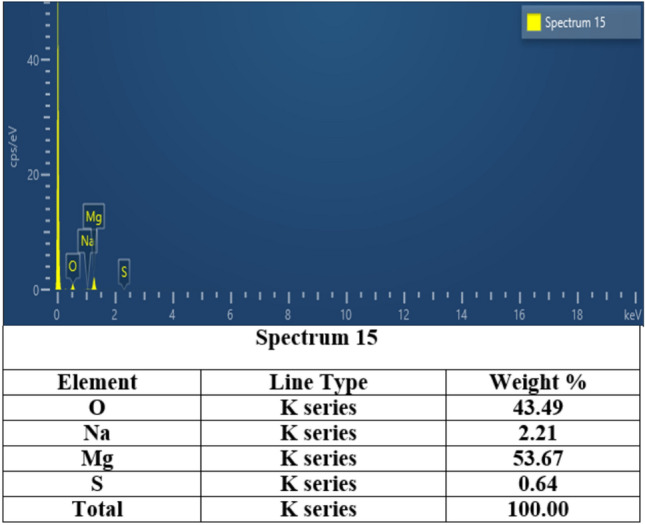
EDX analysis of MgO nano-catalyst.

As indicated in Fig. 1, the scanning electron microscope (SEM) study was carried out at 50 μm magnifications. The generated MgO nano-catalyst typically consists of irregularly shaped particles with active sites, and porous structures, and according to the SEM pictures, is porous in nature. Thus, the catalyst revealed a greater surface area for reaction due to the presence of particles of varying sizes and shapes. Nano MgO EDX spectra were recorded (Fig. 2) to further verify the surface treatment. The EDX spectrum of nano MgO shows the existence of magnesium, oxygen, and other impurities, indicating that an appropriate change has happened. Also, it could be noticed from Fig. 2 that there is a homogeneous distribution of magnesium, and oxygen in the nanocomposite.

#### The Brunauer–Emmett–Teller surface area

The specific surface area of the nano-catalyst particles was determined using Brunauer–Emmett–Teller (BET). It was discovered that the MgO nano-catalyst has a specific surface area of roughly 60.2 m^2^/g, compared to the MgO particles' surface area of 19 m^2^/g^15^ The nano MgO has a bigger surface area than MgO particles, which suggests that it has more active sites available to catalyse the transesterification reaction. Greater biodiesel yield and quicker response times could result from this.

#### Thermogravimetric analysis

**Fig. 3:**
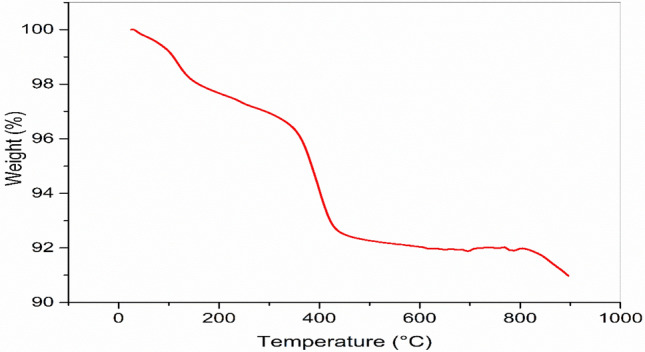
Thermogravimetric analysis for synthesized MgO nano-catalyst.

Thermogravimetric analysis was used to determine the thermal stability and decomposition profiles of MgO and is shown in Fig. 3. The weight of the catalyst decreased with a very little slope as the temperature increased. Possible causes for this weight loss include the catalyst's moisture content. At temperatures between 50 and 150 °C, the water that has been absorbed evaporates. At 305 °C, the catalyst's weight dropped by 4% and reached 96% of its starting value. This weight reduction was maintained throughout. The sample structure was primarily broken down between 300 and 400 °C after this temperature, as seen by the sharp increase in the diagram's slope. The MgO nanocatalyst began to deteriorate at this temperature, which is also known as its thermal breakdown temperature. Higher temperatures may damage the sample structure, so it's essential to know the decomposition temperature, which shows the temperature range in which the material can be used without decomposing. At a temperature of around 800 °C or more, total disintegration of the MgO nano-catalyst was noticed. The TGA curve demonstrated that the MgO nano-catalyst's has excellent thermal stability.

#### X-ray powder diffraction analysis

**Fig. 4:**
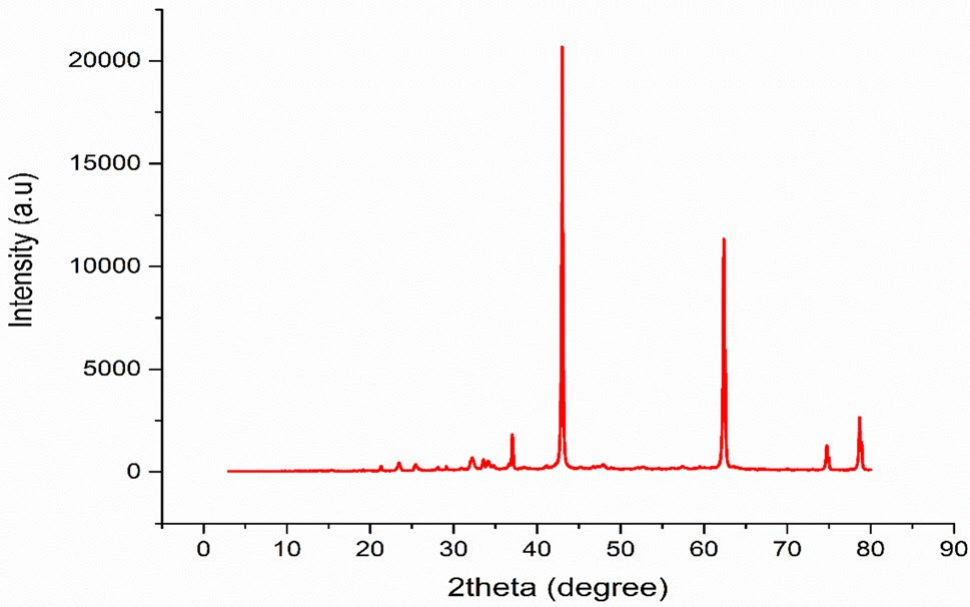
XRD analysis for synthesized MgO nano-catalyst.

As can be seen in Fig. 4, the sharp spectra revealed high crystallinity of the powder. The sharp peaks were visible at 2θ angles at 36.985°, 42.969°, 62.391°, 74.797°, and 78.746°, which correspond to the (111), (200), (220), (311), (222), and (400) planes of magnesium hydroxide with a simple hexagonal phase structure and are in good agreement with the standard JCPDS card number: 01–079-9866. The crystallite size diameter (D) in nanometers of the MgO nanoparticle was calculated by using Debye Scherrer equation (D = K λ / β cos θ) where, D is the average crystal size , λ is the wavelength of the CuKα-radiation , β is the full width at half maximum (FWHM), and θ is the angle of diffraction^16^, and as it was seen in Table 3, the particle size of the synthesised MgO lies between 65.38 nm and 69.78 nm, with a mean crystal size of 67.125 nm.

**Table 3:** Results of XRD pattern for synthesized MgO nano-catalyst.

Two theta value (Degree)	K-constant	$\beta -beta$ FWMH (Rad)	λ-X-ray Wavelength (nm)	Intensity (a.u )	D-(size) (nm)
44.092	0.94	0.0023	0.154	20,686	67.9
63.399	0.94	0.0026	0.154	11,342	65.44
75.478	0.94	0.0028	0.154	1301	65.38
79.594	0.94	0.0027	0.154	2567	69.78

### Optimization of reaction parameters

Biodiesel production reaction conditions were studied by adjusting the following three variables: reaction temperature, reaction time, and the mole ratio of methanol to oil. Accordingly, 17 experiments were devised, with the amount of biodiesel produced serving as the response variable. The expected yield of biodiesel production under various experimental conditions is listed in Table 4. Following an analysis of the experimental data, the following regression equations were obtained in terms of coded factors to predict yield values:

3 $${\text{Yield }} = { 79}.{88} - {3}.{\text{17 A}}^{{2}} - {6}.{\text{61B}}^{{2}} {-}{ 6}.{\text{7C}}^{{2}}$$

**Table 4:** Experimental and predicted responses for the biodiesel production.

Run	Factor 1 A:M/O mole/mole	Factor 2 B:Temp ^°^C	Factor 3 C:Time Min	Experimental yield g/g	Predicted
1	8	70	60	0.8021	0.7997
2	10	50	120	0.8152	0.8341
3	6	50	60	0.9029	0.8341
4	6	50	120	0.8154	0.8341
5	8	30	60	0.8222	0.7997
6	8	50	90	0.8027	0.7988
7	6	30	90	0.715	0.701
8	8	50	90	0.7721	0.7988
9	8	50	90	0.8032	0.7988
10	8	50	90	0.8132	0.7988
11	10	70	90	0.7058	0.701
12	8	70	120	0.7704	0.7997
13	6	70	90	0.6608	0.701
14	8	50	90	0.8029	0.7988
15	10	30	90	0.7222	0.701
16	8	30	120	0.8039	0.7997
17	10	50	60	0.8026	0.8341

**Fig. 5:**
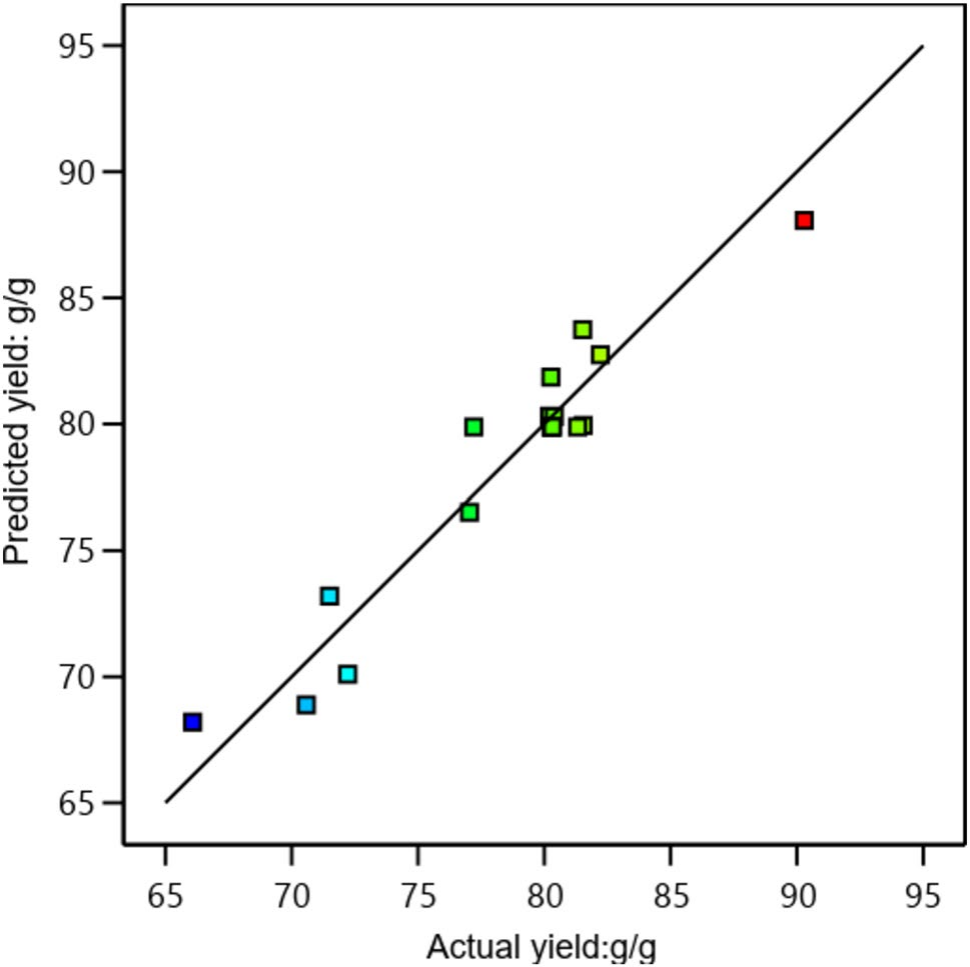
Actual versus predicted biodiesel yield.

Figure 5 demonstrates that the expected values for the yield of biodiesel production responses are quite like the actual values, demonstrating the consistency of the models used for establishing a relationship between the independent process variables and the responses. ANOVA was used to evaluate the statistical significance of the yield model (Table 4). The predicted model's F-value was 9.09, as shown in Table 5, indicating that the model is significant. The model's pure error had a poor correlation, as shown by the "Lack of Fit F-values" of 4.16. The quadratic model fits the data well because the R-squared value for the model Eq. (92) was higher than 0.75^17^.

**Table 5:** ANOVA results for yield response through the RSM modeling.

Source	Sum of squares	df	Mean square	F-value	*p*-value	
Model	467.53	9	51.95	9.09	0.0041	Significant
A-M/O	2.92	1	2.92	0.5103	0.4981	
B-Temp	19.28	1	19.28	3.37	0.1088	
C-Time	19.50	1	19.50	3.41	0.1072	
AB	3.57	1	3.57	0.6251	0.4551	
AC	25.05	1	25.05	4.38	0.0746	
BC	0.4489	1	0.4489	0.0786	0.7874	
A^2^	42.44	1	42.44	7.43	0.0295	
B^2^	184.09	1	184.09	32.22	0.0008	
C^2^	188.74	1	188.74	33.03	0.0007	
Residual	40.00	7	5.71			
Lack of Fit	30.28	3	10.09	4.16	0.1012	Not significant
Pure Error	9.72	4	2.43			
Cor Total	507.53	16				

#### Effect of process variables

**Fig. 6:**
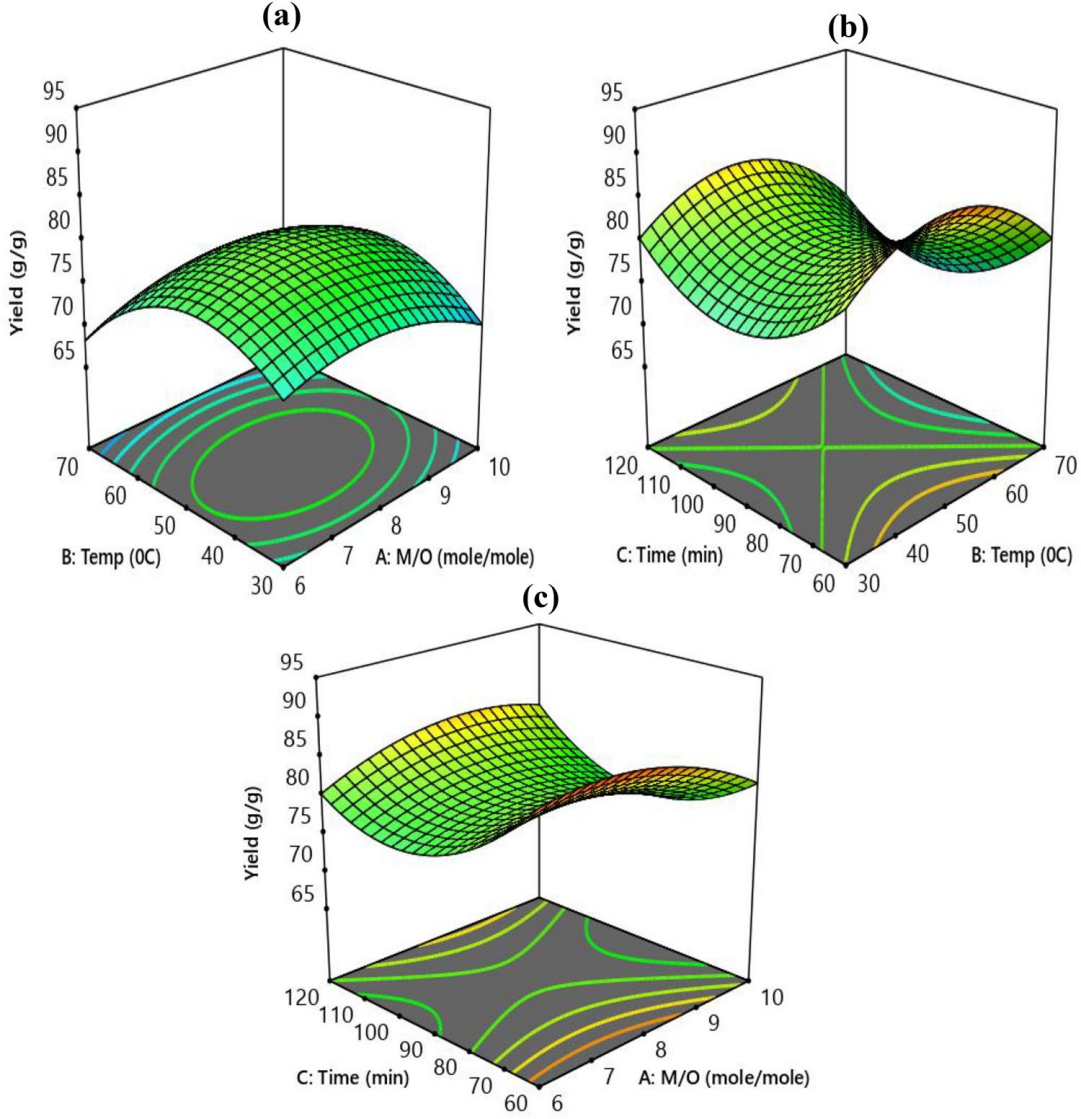
The response surface plot for the effect of temperature and M/O ratio (**a**), time and temperature (**b**), time and M/O ratio (**c**) on biodiesel yield.

The interaction effect of the process variable on the yield is shown by the 3-D response surface in Fig. 6. The contour plot's form can be used to forecast the kinds and intensities of interactions between two independent variables. A major interaction is shown by an elliptical contour plot, whereas a little effect is shown by a circular contour plot. In general, the temperature increases with the biodiesel yield^14^ .

- Effect of oil to methanol molar ratio

Oil to methanol molar ratio has a significant impact on the biodiesel yield. Yields of biodiesel improve with increasing molar ratio and duration. Figure 6 illustrates how the percentage of biodiesel yields increased along with the molar ratio of oil to methanol, which went from 1:6 to 1:10. The best oil to methanol molar ratio for an 88% biodiesel output was determined in this investigation to be 1:7.

- Effect of reaction temperature

The manufacture of biodiesel is significantly impacted by temperature. Raising the temperature above 50 °C causes the methanol to evaporate more quickly, which causes the methanol to be removed from the reaction system and reduces the yield^18^. At the ideal temperature of 50 °C, the largest production of biodiesel was produced, with a maximum yield of 88%.

- Effect of reaction time

Figure 6 illustrates how reaction time affects the transesterification process. At a response time of 60 min, the maximum biodiesel yield was achieved. Before reaching equilibrium, the forward reaction or the biodiesel production moved quickly. However, the backward reaction begins when reactions continue longer than the recommended reaction time. As a result, a longer reaction time lowers the output of biodiesel^14^.

#### Validation of optimized reaction conditions

The validity of the optimized independent parameters, including reaction temperature, reaction time, and methanol/oil ratio, was assessed by comparing the predicted and observed results. The gap between the anticipated and actual response was determined to be below 1.5%, as evidenced in Table 6. This exemplifies the efficacy of the response surface methodology in accurately predicting experimental outcomes. The models generated in this study were precise and reliable^19^. The optimum values for the biodiesel production were as follows: time, 60 min; temperature, 50 ^°^C; methanol to oil ratio, 7; and a maximum yield of 0.89 g/g.

**Table 6:** Model validation at optimum conditions for the biodiesel production.

Optimum conditions	
Time (min)	60
Temperature (^0^C)	50
Methanol to oil ratio (mole/mole)	7
Predicted Yield (g/g)	89
Experimental Yield (g/g)	88
% Error	1.12

### Biodiesel characterization

#### Fourier transform infrared (FTIR) spectroscopy

**Fig. 7:**
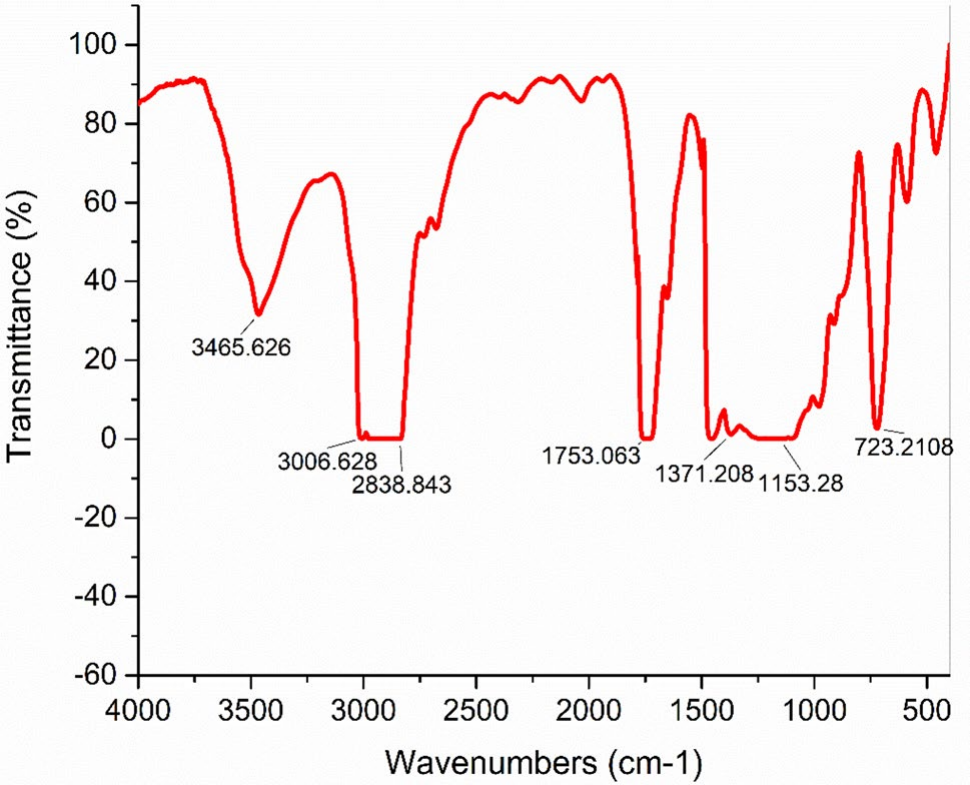
FTIR spectrum for the biodiesel produced.

For monitoring WCO's transesterification processes, the FTIR spectra of biodiesel was used. According to Fig. 7, the carbonyl (C = O) at 1753.06 cm^-1^ and the stretching vibration of (C-O) from the ester at 1371.2–1153.3 cm^-1^ are particularly strong absorption bands for the ester group. Other functional group peaks in biodiesel, like stretching vibration of CH_3_ at 2838.8 and bending vibration (CH_2_)_n_ at 723.2 cm^-1^, are seen in the spectrum. O–H bands at 3006.6 cm^-1^ confirmed the presence of FFA and glycerol O–H groups in the biodiesel ^20–23^.

#### Gas chromatography-mass spectrometry

**Fig. 8:**
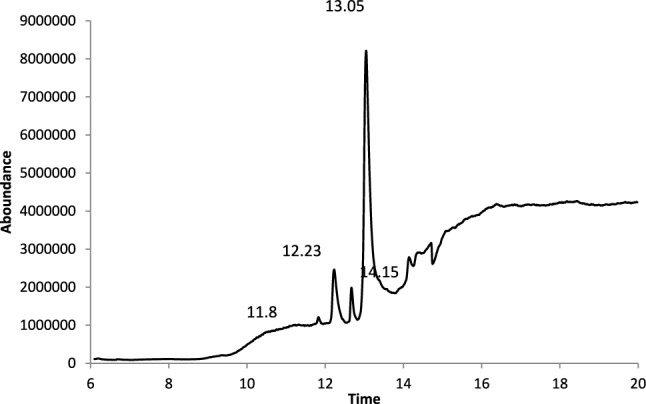
GC–MS analysis for the biodiesel produced.

The analysis of the GC data, as shown in Fig. 8 and summarized in Table 7, provided valuable insights into the retention duration and fragmentation pattern. This analysis highlighted the presence of seven distinct FAME peaks, each exhibiting unique characteristics. Tariq et al.^22^ conducted a study to determine the FAME composition and the corresponding common names found in biodiesel under ideal circumstances. The findings are presented in Fig. 8, confirming the occurrence of methyl ester formation in most triglycerides, indicating the production of biodiesel. Because of the composition of the initial waste cooking oil, there are a lot of saturated free fatty acid esters. Furthermore, the biodiesel GC–MS chromatogram data show that good quantities of methyl esters were created, and the high yield of the transesterification reaction was confirmed by the modest amounts of glycerin and methanol as impurities, as well as mono and diglycerides as intermediates.

**Table 7:** GC analysis results of the biodiesel.

RT	Area Pct	Compound
11.8259	16.3211	9,17-Octadecadienal, (Z)- methyl ester
12.2321	8.8216	n-Hexadecanoic acid, methyl ester
12.6727	3.1047	10,13-Octadecadienoic acid, methyl ester
13.0504	57.5306	9,12-Octadecadienoic acid (Z,Z)-, methyl ester
14.149	3.5005	11,13-Eicosadienoic acid, methyl ester
14.4179	4.7563	8,11-Eicosadienoic acid
14.704	5.9651	11,13-Eicosadienoic acid, methyl ester

Table 7 presents the primary constituents of the methyl ester compound, which comprise 9,17-Octadecadienal, (Z)- methyl ester (16.3%), 9, 12-Octadecadienoic acid (Z, Z), methyl (57.5%), and n-Hexadecenoic acid, methyl ester (8.8%). The results of the GC–MS spectrum analysis indicate that waste cooking oil exhibits a high potential for conversion into fatty acid methyl esters (FAME), making it a viable option for large-scale biodiesel production. According to Table 7 of the gas chromatography (GC) analysis, the fatty acid methyl ester (FAME) content was approximately 95.3% by weight, and the highest mass yield of biodiesel produced was 92%. The conversion efficiency, as determined by Eq. (3), is expected to be 88 weight percent. Table 8 displays the characterization outcomes of the biodiesel produced, in comparison to established benchmarks.

**Table 8:** Characteristics of the produced biodiesel, compared with standard values.

Property	Test method ASTM	ASTM D6751, limit for B100	Test result
Density at 15 °C, Kg/m^3^	D4052	860–900	898.1
Flash Point, °C	D93	Min. 93	90.1
Cloud point, °C	D2500	− 12	− 6.8
Heating value, Cal/g	D240	8907.7	9241.65

## Conclusion

The process of catalytic transesterification of waste cooking oil (WCO) was investigated for the purpose of biodiesel production. The catalyst employed in this study is magnesium oxide (MgO). The synthesis of a magnesium oxide (MgO) nano-catalyst was conducted using the sol–gel process, followed by a subsequent calcination procedure. The catalyst that was obtained displayed an average particle size of 67.125 nm, exhibited remarkable thermal stability, and possessed a significant specific surface area of 60.2 m^2^/g. The study determined that the most favorable reaction conditions involved a molar ratio of 1:7 between waste cooking oil (WCO) and methanol, a concentration of 1 wt.% for the magnesium oxide (MgO) nano catalyst, a reaction temperature of 50 °C, and a reaction duration of 60 min. The study determined the FAME content, maximum yield of biodiesel, and conversion efficiency percentage to be 95.3%, 92%, and 88 wt.% respectively. The empirical validation experiments yielded results that demonstrated a strong agreement with the theoretical predictions, as the observed outcomes deviated by less than 1.5% from the expected outcomes. The biodiesel produced was subjected to analysis, and its composition was assessed using gas chromatography–mass spectrometry and Fourier transform infrared spectroscopy techniques. The biodiesel that was produced exhibited satisfactory adherence to the specified criteria when analyzed according to the American standard (ASTM D6571) for the assessment of its diverse properties.

## Data Availability

All data generated during this study are included in this published article.

## Abbreviations

ASTM: American Society for Testing and Material
B100: Biodiesel 100%
BET: Brunauer–Emmett–Teller specific surface area analysis
FFA: Free Fatty Acid
FAME: Fatty acid methyl ester
FTIR: Fourier transform infrared spectroscopy
M/O: Methanol to oil
RSM: Response surface methodology
RT: Retention time
SEM –EDX: Scanning electron microscope coupled with energy dispersive X-ray scattering scanning analysis
TGA: Thermo-Gravimetric analysis
XRD: X-ray powder diffraction
WCO: Waste cooking oil
